# PD-L1 expression in lung adenosquamous carcinomas compared with the more common variants of non-small cell lung cancer

**DOI:** 10.1038/srep46209

**Published:** 2017-04-07

**Authors:** Xiaohua Shi, Shafei Wu, Jian Sun, Yuanyuan Liu, Xuan Zeng, Zhiyong Liang

**Affiliations:** 1 Peking Union Medical College Hospital, Department of Pathology, Beijing, 100730, China

## Abstract

Lung adenosquamous cell carcinomas (ASCs) is a rare variant of NSCLC with a poorer prognosis and fewer treatment option than the more common variants. PD-L1 expression is reported to be the predictor of clinical response in trials of NSCLC. In our study, PD-L1 expression was evaluated via immunohistochemistry using a specific monoclonal antibody (SP263), and PD-L1 mRNA expression was evaluated via *in situ* hybridization. This study included 51 ASCs, 133 lung adenocarcinomas, and 83 lung squamous cell carcinomas (SCC). Similar results were obtained for PD-L1 expression measured at the mRNA and protein level (*k* coefficient, 0.851, P = 1.000). PD-L1 expression was significantly higher in the squamous versus glandular component of the 36 ASCs in which the components were analyzed separately. The PD-L1 expression rate was similar in the squamous cell component of ASCs and lung SCC (38.89% vs. 28.92%, P = 0.293), so does the adenocarcinoma component of ASCs and lung adenocarcinomas (11.11% vs 13.53%, P = 1.000). PD-L1 expression correlated significantly with lymphovascular invasion (P = 0.016), but not with *EGFR, KRAS*, and *ALK* mutations in lung ASCs. Anit-PD-L1 is a promising treatment option in lung ASC cases in which PD-L1 upregulated and *EGFR* mutations are present.

Lung adenosquamous cell carcinoma (ASC) is a rare variant of non-small cell lung cancer (NSCLC), whose prognosis is worse than that of the more common variants^1^. Platinum-based chemotherapy is the mainstay treatment for ASC. However, new strategies are emerging. These include personalized therapies [e.g., administration of tyrosine kinase or anaplastic lymphoma kinase (ALK) inhibitors] based on genetic alterations in tumor cells in patients with lung adenocarcinomas^2^, and immune checkpoint blockade therapies, which have achieved promising results in NSCLC as well as melanoma and renal cell carcinoma patients^3^,^4^. Recently, FDA in US approved pembrolizumab, which is a programmed death 1 (PD1) antibody, for the treatment of certain patients with metastatic NSCLC.

Immune checkpoints maintain self-tolerance and protect peripheral tissues by inhibiting immune responses^5^. Key checkpoint proteins include PD-1 and its ligand PD-L1, which are expressed by both inflammatory cells and tumor cells in peripheral tissues. In healthy individuals, PD-1 and PD-L1 inhibit T cell activity to prevent autoimmune responses; when overexpressed in tumor cells, they allow tumor cells to escape T cell-mediated antitumor immune responses and immune surveillance^6^.

Targeted disruption of PD-L1 function has been shown to improve outcomes in NSCLC. For example, the clinical results of KEYNOTE-024 showed that pembrolizumab was superior compared to chemotherapy for both the primary endpoint of progression-free survival (PFS), and the secondary endpoint of overall survival (OS)^7^. And in a previous study, approximately 20% of unsorted NSCLC patients receiving anti-PD-1/PD-L1 antibodies had a meaningful response to therapy^3^,^4^. Careful selection of patients who will benefit from this targeted therapy is urgently required. Herein, we determined the expression pattern of PD-L1 in lung ASCs and the association between PD-L1 expression and 1) various clinicopathological characteristics and 2) alterations in major driver oncogenes including EGFR, KRAS, and ALK.

Identification of the patients who will benefit from anti-PD1/PD-L1-targeted therapy is essential; doing so also spares those who will not benefit from unnecessary treatments and reduces the occurrence of side effects resulting from loss of PD-1/PD-L1 inhibiting function and consequent activation of the autoimmune system. Hence, a reliable detection system is needed, and toward this goal, we compared PD-L1 expression determined via two different methods: immunohistochemistry (IHC) and RNA *in situ* hybridization (ISH).

## Results

### Comparison of PD-L1 expression determined via IHC and RNA ISH

**Figure 1:**
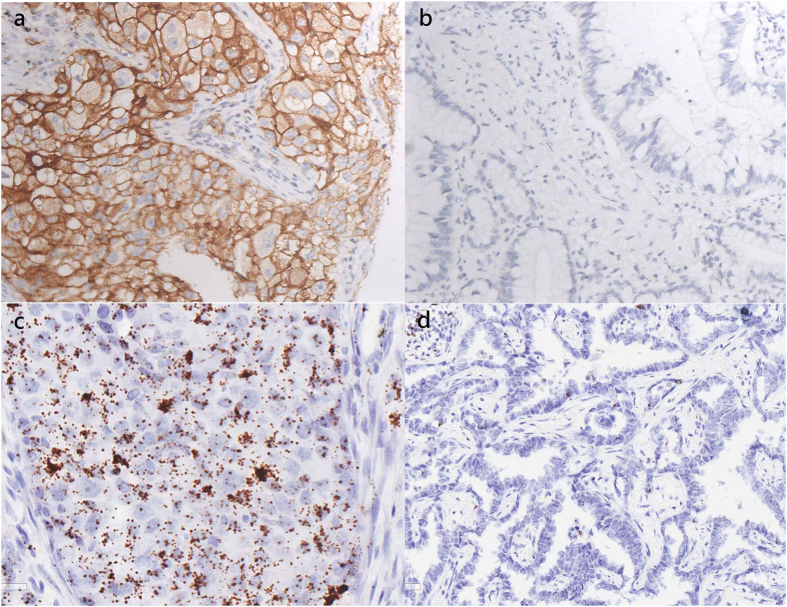
Representative results of PD-L1 expression in lung ASC. (**a**) Positive results of PD-L1 examined via immunohistochemistry method. (x100) (**b**) Negative results of PD-L1 examined via immunohistochemistry method. (x100) (**c**) Positive results of PD-L1 examined via RNA *in situ* hybridization method. (x100) (**d**) Negative results of PD-L1 examined via RNA *in situ* hybridization method. (x100).

Of the 51 lung ASCs in this study, 36 had separable glandular and squamous components that were evaluated independently. Consequently, a total of 87 tissue samples were examined. PD-L1 expression was detected by IHC and ISH in 39.22% and 37.25% of the lung ASCs, respectively. There was no differences between the two techniques in terms of which tumors were negative and which were positive. Representative IHC and RNA ISH results are shown in Fig. 1. The agreement between the IHC and mRNA ISH results was nearly perfect (91.3%, 179/196), with a κ –coefficient of 0.851. There was no significant difference between the two methods according to the McNemar-Bowker test (P = 1.000) (Table 1).

### PD-L1 expression in lung ASCs compared with the more common NSCLC variants

As determined via IHC, PD-L1 was expressed in 39.22% of lung ASCs. When the two components of the 36 lung ASCs were examined separately, the PD-L1 expression rate differed significantly (P = 0.017): 11.11% in the glandular component and 38.89% in the squamous component. In lung adenocarcinomas (n = 133), the PD-L1 expression rate was 13.53%, which was similar to that of the glandular component of lung ASCs (P = 1.000) (Table 2). In lung squamous cell carcinomas (n = 83), the PD-L1 expression rate was 28.92%, which approximated that of the squamous component of lung ASCs (P = 0.293) (Table 3).

### Comparison of PD-L1 expression with clinicopathological characteristics

Among the 51 patients with lung ASCs, 33 were men, 18 were women, 22 were smokers, and 29 were non-smokers. The median age at diagnosis was 61.14 years (range: 34–80). The average tumor size were 4.47 cm (range: 2–10 cm). The tumors were stage I, II, III, and IV in 12 (23.5%), 6 (11.8%), 29 (56.9%) and 4 (7.8%) cases, respectively.

The growth pattern of the glandular component of the lung ASCs was classified as lepidic (4 tumors), acinar (40 tumors), or other type including mucinous (1 tumor), solid (4 tumors), and micropapillary (2 tumors). The differentiation status of the squamous component was classified as well (9 tumors), moderate (27 tumors), or poor (15 tumors). Lymphovascular invasion, pleural involvement, and positive node metastasis were observed in 32, 32, and 39 cases, respectively. There was no significant association between PD-L1 expression and most of the clinicopathological characteristics (sex, age, smoking, tumor size, lymph node metastasis, invasion of pleura, recurrence, or death). Only lymphovascular invasion correlated significantly with overexpression of PD-L1 in lung ASCs (P = 0.016) (Table 4).

**Figure 2:**
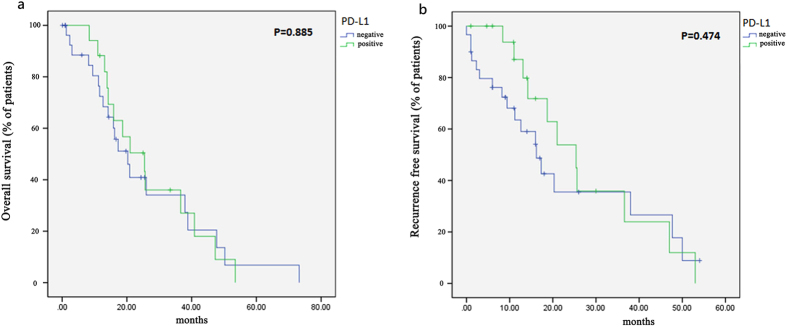
Kaplan–Meier survival curves. (**a**) The was no significant difference regarding overall survival between PD-L1 positive and negative group. (**b**) The was no significant difference regarding recurrence free survival between PD-L1 positive and negative group.

After surgery, 29 of 49 patients received adjuvant therapy, including chemotherapy (28 patients), radiation therapy (10 patients), or both (9 patients). None of them received anti PD-1 or PD-L1 targeted therapy. Survival data were available for 49 of the 51 lung ASC patients (three patients were further lost to follow-up after disease recurrence). Tumors recurred or metastasized in 20 (40.82%) of the 49 patients at an average time of 14.03 months (range: 1–54 months) after surgery. Twelve (25.53%) of 47 patients died within an average time of 14.89 months (range: 0–33 months) after surgery. At the end of the follow-up period, 22 patients were alive without disease. There were no association between either OS or RFS and PD-L1 protein expression (P = 0.885 and 0.474, respectively) (Fig. 2).

### Comparison of PD-L1 expression with alterations in major driver oncogenes

The EGFR mutation rate in lung ASCs was 57.45%, which the glandular and squamous component show convergent results in 34 of 36 cases. The KRAS mutation and ALK rearrangement rates were 6.38% and 5.89%, respectively, with identical results for both components. PD-L1 expression was similar in lung ASCs with EGFR mutations, KRAS mutations, or ALK rearrangements (P = 1.000, 0.544, and 0.553, respectively) (Table 5).

## Discussion

Therapy targets are primarily based on gene mutations or rearrangements; however, most lung cancers lack an actionable gene aberration. Thus, targets unrelated to genetic alterations are needed. It is fortunate that clinical trials of anti-PD-1/PD-L1-based therapies have shown encouraging results. For example, comparing with platinum-based chemotherapy, pembrolizumab achieved better effect in patients with advanced NSCLC and PD-L1 expression on at least 50% of tumor cells^7^,^8^. Hence, PD-1 and PD-L1 are potential targets in lung cancers.

Preclinical trials of immune check point inhibitors are designed to treat NSCLC patients regardless of tumor histological type. Hence, the expression rate of PD1/PD-L1 in a particular variant is based on a relatively small sample number in these trials, and determination of the differences (if any) between the variants requires further exploration. There is little information about PD-L1 expression in lung ASCs. Our study evaluated PD-L1 expression in 51 lung ASCs, 133 lung adenocarcinomas, and 83 lung squamous cell carcinomas, and showed expression rates of 39.22%, 28.92%, and 13.53%, respectively. In 36 lung ASCs, the glandular and squamous components were separately examined. The PD-L1 expression rate was almost the same in the glandular component and lung adenocarcinomas (11.11% vs. 13.53%, P = 1.000) and was similar in the squamous component and lung squamous cell carcinomas (38.89% vs. 28.92%, P = 0.293). It differed significantly between the two components of lung ASC (P = 0.017), and we are the first to report this difference.

In our study, we found out that PD-L1 expression is not only higher in the squamous component of lung ASC comparing with the glandular component, but also higher in squamous cell carcinoma than in lung adenocarcinoma. In the Yale cohort study, lung squamous cell carcinomas had higher PD-L1 levels than did lung adenocarcinomas (56.7% vs. 27.5%, P = 0.009), which was similar with our study^9^. Given the relatively high expression rate of PD-L1 in squamous cell carcinomas, it is not surprising that NSCLCs with a squamous histology tended to have better response rates (e.g., a 6-month progression-free survival rate) in clinical trials of PD-1/PD-L1 or PD-L2-targeted immunotherapy than did those with a non-squamous histology^10^,^11^. The Food and Drug Administration recently approved an anti-PD-1 single-agent therapy for patients who have progressed after standard chemotherapy for advanced (metastatic) head and neck squamous cell carcinoma.

Because lung ASCs consist of both glandular and squamous components, there are two opinions regarding their origin: monoclonal or polyclonal. Most research supports the monoclonal theory owing to the results of examinations of mutations in the major driver oncogenes and X chromosome analyses^12^,^13^. For example, we previously found identical driver oncogene mutations in the glandular and squamous components of lung ASCs^14^. Others showed that down regulation of yes-associated protein promoted the transition of lung adenocarcinomas to squamous cell carcinomas in liver kinase B1-deficient mice^15^. As shown in the present study, PD-L1 expression differed significantly between the two components, with a much higher expression rate in the squamous component. Based on the above evidence, we speculate that gain of PD-L1 expression during phenotype transdifferentiation from adenocarcinoma to squamous cell carcinoma in lung ASC helps tumor cells escape immune surveillance, consequently resulting in worse prognosis.

In our study, PD-L1 expression did not significantly correlate with most of the clinicopathological characteristics of lung ASC or with mutations in driver oncogenes such as EGFR, KRAS, and ALK. To data, there were no detail information regarding EGFR mutation and lung ASC. Most of them focusing on the association of EGFR and lung adenocarcinoma or pan-NSCLC. In a previous meta-analysis positive PD-L1 expression correlated with poor prognosis in NSCLC patients, whereas there was no correlation between PD-L1 expression and clinical features (sex, histology, smoking status, tumor stage, and lymph node metastasis)^16^. Others showed a link between activation of epidermal growth factor receptor (EGFR) signaling and upregulation of PD-L1, PD-1, and CTLA-4 in an EGFR-driven murine model of lung cancer and human NSCLC cell lines^17^ and between activating EGFR mutations and PD-L1 overexpression in tumors^18^. While Mu *et al*. observed no significant correlation between PD-L1 expression and EGFR/ALK status in stage I NSCLC patients^19^. Rather, we found that PD-L1 expression was independent of several types of gene mutations and could be expressed in lung ASCs with EGFR mutations.

Some investigators believe that constitutive PD-L1 expression on cancer cells is a secondary reaction to oncogenic stress^20^. In clinical trials, PD-L1-positive patients receiving EGFR tyrosine kinase inhibitors (TKIs) had higher response rates and longer time to progression than did PD-L1-negative patients^21^. An ongoing clinical trial is evaluating the therapeutic effects of a combination treatment consisting of nivolumab and erlotinib (NCT01454102) in NSCLCs with EGFR mutations, which represent a minority of cases. As shown here, PD-L1 expression can be detected in ASCs with EGFR mutations, and we suggest that immune checkpoint therapy combined with administration of an EGFR TKI may effectively treat these rare tumors.

A prerequisite for precise targeted therapy is a suitable diagnostic method that allows determination of the expression rate of PD-L1 in lung tumors. Immunohistochemical tests are easily performed, relatively inexpensive, and widely accessible. There are several commercial antibodies for detecting PD-L1 including the SP263 and SP142 clones (Ventana) and 28–8 and 22C3 clones (Dako, Carpinteria, CA, USA), which correspond to different clinical drugs and have different positive cutoff values. A consensus detection method and evaluation criteria are needed for routine selection of suitable patients for targeted treatments with these antibodies in the future. According to the central dogma of genetics, genes are first transcribed into mRNAs, which are then translated into proteins. In the present study, PD-L1 expression was assessed at the RNA level via mRNA ISH and at the protein level via IHC. The concordance rate of the two methods was nearly 100%. More data evaluating detection methods and clinical treatment outcomes are needed.

As a study limitation, we evaluated PD-L1 expression in tumor cells, but not tumor-infiltrating cells, which are thought to modulate the response to anti-PD-L1 therapy. We did not do so mainly because the amount of the TMA tissues used in our study was limited and was not guaranteed by all the cores in the tumor infiltration lymphocytes. In the future, we will overcome this problem by evaluating whole tissue sections instead of TMA samples.

In conclusion, PD-L1 expression differs between the two components of lung ASCs. Given the complexity of lung ASCs, their treatment outcomes may be improved by administration of both EGFR TKIs and anti-PD-1/PD-L1 antibodies in cases where EGFR mutations are present and PD-L1 is overexpressed. We suggest that anti-PD-L1 therapy is a promising option for lung ASC patients meeting these criteria.

## Methods

### Patient information

Fifty-one patients who underwent surgery with a final diagnosis of lung ASC at Peking Union Medical College Hospital (Beijing, China) between January, 2010 and December, 2014 were enrolled in this study. All final diagnoses were based on the morphology of tumor samples stained with hematoxylin and eosin (H&E). In cases of poorly differentiated tumors or histologically atypical adenocarcinoma or squamous components, immunostaining for p63, p40, napsin A, and thyroid transcription factor 1 was also performed to confirm the final results. To assess PD-L1 expression in the common variants of NSCLC, 133 patients with lung adenocarcinoma and 83 patients with lung squamous cell carcinoma were also enrolled in this study.

Clinical information including patient age, sex, and smoking habits, clinical stage, treatment methods, relapse-free survival (RFS) time, and overall survival (OS) time were collected from the clinical archives. Clinical stage was determined at the time of surgery in accordance with the tumor-node-metastasis staging system outlined in the seventh edition of the American Joint Commission on Cancer. RFS was defined as the time from surgery to relapse or the endpoint of the study. Overall survival (OS) was defined as the time from surgery to death or the endpoint of the study.

All H&E slides for each tumor sample were re-reviewed by two experienced pathologists to confirm the final diagnosis, the growth pattern of the glandular components and the differentiation of the squamous components of lung ASCs were recorded. Neither pathologists was aware of the clinical information or PD-L1 expression results during the review. The following pathological characteristics were also collected during the review of the HE slides: tumor size, lymphovascular invasion, pleura invasion, and node metastasis status.

This study was approved by the Institutional Review Board of Peking Union Medical College Hospital. An informed consent was obtained from all patients. The methods were carried out in accordance with the approved guidelines.

### Tissue microarray (TMA) construction

Morphologically representative areas on the H&E slides were selected, and the corresponding formalin-fixed, paraffin-embedded (FFPE) primary tumor specimens were obtained from the Department of Pathology. A TMA construction system (Quick-Ray UT-06, UNITMA) was used. Three core tissue biopsies, each 1.0 mm in diameter, were collected for each case. TMAs were prepared for lung adenocarcinoma, lung squamous cell carcinoma, and lung ASC. The glandular and squamous components of lung ASCs, which were distinguishable on the H&E slides, were collected separately for construction of the lung ASC TMA.

### PD-L1 detection via IHC

IHC was performed on FFPE TMA sections with a thickness of 4 μm, using standard staining protocols and a Ventana Benchmark XT autostainer (Ventana Medical Systems, Inc., Tucson, AZ, USA). The anti-PD-L1 antibody SP263 (Ventana) was used for immunostaining. A tumor was considered PD-L1-positive if membrane staining was apparent in more than 25% of the cells in the tumor sample, as described by Ratcliffe *et al*.^22^.

### PD-L1 mRNA detection via ISH

For *in situ* detection of PD-L1 mRNA, an RNAscope 2.0 HD detection kit (Brown) (Advanced Cell Diagnostics, Hayward, CA, USA) was used according to the manufacturer’s instructions. Briefly, 2- to 3-mm-thick FFPE tissue sections were deparaffinized, heated, protease-treated, and hybridized with a PD-L1 probe (#312351) at 40 °C for 2 h. After washing and amplification, the target RNA was detected by staining with 3, 39-diaminobenzidine. Nuclei were counterstained with hematoxylin. Positive staining was indicated by the presence of brown punctuate dots in the cytoplasm or nucleus. Separate dots were counted and scored as follows: 1–10 dots, 1; 10–15 dots, 2; 15–20 dots, 3; and more than 20 dots, 4. Tumors with a score of 3 or 4 were considered positive for PD-L1 expression.

### Detection of mutations in major driver oncogenes

The mutation profiles of EGFR and KRAS were examined by using a Human EGFR Gene Mutations detection kit (Real-time Fluorescent PCR) and a Human KRAS Gene Mutations detection kit (Beijing ACCB Biotech, Beijing, China), respectively. In total, the tests examined 63 hotspot mutations including 45 in exons 18, 19, 20, and 21 of EGFR, and 12 in exons 2 and 3 of KRAS. Quantitative polymerase chain reaction (PCR) was performed by using an Mx3000 P PCR system (Agilent, Santa Clara, CA, USA) with the following settings: 95 °C for 10 min, 40 cycles of 95 °C for 15 s, and 60 °C for 1 min. The results were interpreted per the manufacturers’ instructions. ALK rearrangement was examined via fluorescence *in situ* hybridization using a break-apart probe for the ALK gene (Vysis LSI ALK Dual Color; Abbott Molecular, Abbott Park, IL, USA).

### Statistical analysis

Binary correlative variables were evaluated by using Fisher’s exact test. RFS and OS were calculated by using the Kaplan-Meier method. All tests were two-sided. P values < 0.05 were considered statistically significant. All statistical analyses were performed by using SPSS statistics v20.0.0 software (SPSS Inc., Chicago, IL, USA).

For analysis of the concordance between the results of IHC and RNA ISH, a κ-value was calculated, and the significance of the κ-value was evaluated as follows: ≤0.40, poor to fair concordance; 0.41–0.60, moderate concordance; 0.61–0.80, substantial concordance; and 0.81–1.00, almost perfect concordance.

## Additional Information

**How to cite this article:** Shi, X. *et al*. PD-L1 expression in lung adenosquamous carcinomas compared with the more common variants of non-small cell lung cancer. *Sci. Rep.* **7**, 46209; doi: 10.1038/srep46209 (2017).

**Publisher's note:** Springer Nature remains neutral with regard to jurisdictional claims in published maps and institutional affiliations.

## Figures and Tables

**Table 1:** The relationship of IHC and mRNA ISH examination methods.

		PD-L1 IHC		Kappa	P
		−	+		
PD-L1 RNA ISH	−	63	3	0.851	1.000
	+	2	20		

IHC: immunohistochemistry; ISH, *in situ* hybridization.

**Table 2:** Comparison of PD-L1 expression between AD and the glandular components of lung ASC.

	PD-L1		Total	Expression rate	P
	−	+			
AD	115	18	133	13.53%	1.000
AD of ASC	32	4	36	11.11%	

AD, adenocarcinoma; ASC, adenosquamous cell carcinoma.

**Table 3:** Comparison of PD-L1 expression between SCC and the squamous components of lung ASC.

	PD-L1		Total	Expression rate	P
	−	+			
SCC	59	24	83	28.92%	0.293
SCC of ASC	22	14	36	38.89%	

SCC: squamous cell carcinoma; ASC, adenosquamous cell carcinoma.

**Table 4:** Relationship between PD-L1 IHC expression and Clinicopathological characteristics of lung ASC patients.

Characterisitcs	NO. of patients (Percentage)	PD-L1 IHC		
		+	−	P
No. (Total 51)		20	31	
Age				0.323
>=70	12(23.5%)	3	9	
<70	39(76.5%)	17	22	
Gender				0.371
Female	18(35.3%)	6	12	
Male	33(64.7%)	14	19	
Smoking				1.000
Smokers	22(43.1%)	9	13	
Non- smokers	29(56.9%)	11	18	
Tumor Size (cm)				1.000
>3	35(68.6%)	14	21	
<=3	16(31.4%)	6	10	
Stage				0.565
Early (I–II)	18(35.3%)	6	12	
Advanced (III–IV)	33(64.7%)	14	19	
Invasion of pleura				0.554
YES	32(62.7%)	14	18	
NO	19(37.3%)	6	13	
Node metastasis				0.743
YES	39(76.5%)	16	23	
NO	12(23.5%)	4	8	
Lymphovascular invasion				0.016
YES	32(62.7%)	17	15	
NO	19 (37.3%)	3	16	
Recurrence*				1.000
YES	20(40.8%)	8	12	
NO	29(59.2%)	11	18	
Death**				0.489
YES	12(26.1%)	3	9	
NO	34(73.9%)	14	20	

^*^Two patients lost of follow-up.

^**^Five patients lost of follow-up.

**Table 5:** Comparison of PD-L1 expression with major driver oncogene alterations in lung ASC.

		EGFR		P	K-Ras		P	ALK		P
		−	+		−	+		−	+	
PD-L1	−	13	17	1.000	29	1	0.544	30	1	0.553
	+	7	10		15	2		18	2	

## References

[b1] Gawrychowski J., Brulinski K., Malinowski E. & Papla B. Prognosis and survival after radical resection of primary adenosquamous lung carcinoma. Eur J Cardiothorac Surg 27, 686–92 (2005). 15784375 10.1016/j.ejcts.2004.12.030

[b2] Shea M., Costa D. B. & Rangachari D. Management of advanced non-small cell lung cancers with known mutations or rearrangements: latest evidence and treatment approaches. Ther Adv Respir Dis 10, 113–29 (2016). 26620497 10.1177/1753465815617871 PMC5933559

[b3] Brahmer J. R. . Safety and activity of anti-PD-L1 antibody in patients with advanced cancer. N Engl J Med 366, 2455–65 (2012). 22658128 10.1056/NEJMoa1200694 PMC3563263

[b4] Topalian S. L. . Safety, activity, and immune correlates of anti-PD-1 antibody in cancer. N Engl J Med 366, 2443–54 (2012). 22658127 10.1056/NEJMoa1200690 PMC3544539

[b5] Pardoll D. M. The blockade of immune checkpoints in cancer immunotherapy. Nat Rev Cancer 12, 252–64 (2012). 22437870 10.1038/nrc3239 PMC4856023

[b6] Rozali E. N., Hato S. V., Robinson B. W., Lake R. A. & Lesterhuis W. J. Programmed death ligand 2 in cancer-induced immune suppression. Clin Dev Immunol 2012, 656340 (2012). 22611421 10.1155/2012/656340 PMC3350956

[b7] Reck M. . Pembrolizumab versus Chemotherapy for PD-L1-Positive Non-Small-Cell Lung Cancer. N Engl J Med (2016). 10.1056/NEJMoa1606774 27718847

[b8] Herbst R. S. . Pembrolizumab versus docetaxel for previously treated, PD-L1-positive, advanced non-small-cell lung cancer (KEYNOTE-010): a randomised controlled trial. Lancet 387, 1540–50 (2016). 26712084 10.1016/S0140-6736(15)01281-7

[b9] Velcheti V. . Programmed death ligand-1 expression in non-small cell lung cancer. Lab Invest 94, 107–16 (2014). 24217091 10.1038/labinvest.2013.130 PMC6125250

[b10] Brahmer J. R. Harnessing the immune system for the treatment of non-small-cell lung cancer. J Clin Oncol 31, 1021–8 (2013). 23401435 10.1200/JCO.2012.45.8703

[b11] Cooper W. A. . PD-L1 expression is a favorable prognostic factor in early stage non-small cell carcinoma. Lung Cancer 89, 181–8 (2015). 26024796 10.1016/j.lungcan.2015.05.007

[b12] Kang S. M. . Identical epidermal growth factor receptor mutations in adenocarcinomatous and squamous cell carcinomatous components of adenosquamous carcinoma of the lung. Cancer 109, 581–7 (2007). 17186532 10.1002/cncr.22413

[b13] Niho S., Yokose T., Kodama T., Nishiwaki Y. & Mukai K. Clonal analysis of adenosquamous carcinoma of the lung. Jpn J Cancer Res 90, 1244–7 (1999). 10622536 10.1111/j.1349-7006.1999.tb00703.x PMC5926013

[b14] Shi X. . Screening for major driver oncogene alterations in adenosquamous lung carcinoma using PCR coupled with next-generation and Sanger sequencing methods. Sci Rep 6, 22297 (2016). 26923333 10.1038/srep22297 PMC4770439

[b15] Gao Y. . YAP inhibits squamous transdifferentiation of Lkb1-deficient lung adenocarcinoma through ZEB2-dependent DNp63 repression. Nat Commun 5, 4629 (2014). 25115923 10.1038/ncomms5629

[b16] Wang A. . The prognostic value of PD-L1 expression for non-small cell lung cancer patients: a meta-analysis. Eur J Surg Oncol 41, 450–6 (2015). 25682184 10.1016/j.ejso.2015.01.020

[b17] Akbay E. A. . Activation of the PD-1 pathway contributes to immune escape in EGFR-driven lung tumors. Cancer Discov 3, 1355–63 (2013). 24078774 10.1158/2159-8290.CD-13-0310 PMC3864135

[b18] Azuma K. . Association of PD-L1 overexpression with activating EGFR mutations in surgically resected nonsmall-cell lung cancer. Ann Oncol 25, 1935–40 (2014). 25009014 10.1093/annonc/mdu242

[b19] Mu C. Y., Huang J. A., Chen Y., Chen C. & Zhang X. G. High expression of PD-L1 in lung cancer may contribute to poor prognosis and tumor cells immune escape through suppressing tumor infiltrating dendritic cells maturation. Med Oncol 28, 682–8 (2011). 20373055 10.1007/s12032-010-9515-2

[b20] Le D. T. . PD-1 Blockade in Tumors with Mismatch-Repair Deficiency. N Engl J Med 372, 2509–20 (2015). 26028255 10.1056/NEJMoa1500596 PMC4481136

[b21] D’Incecco A. . PD-1 and PD-L1 expression in molecularly selected non-small-cell lung cancer patients. Br J Cancer 112, 95–102 (2015). 25349974 10.1038/bjc.2014.555 PMC4453606

[b22] Marianne J. Ratcliffe . A Comparative Study of PD-L1 Diagnostic Assays and the Classification of Patients as PD-L1 Positive and PD-L1 Negative. In American Association for Cancer Research (AACR) Annual Meeting (New Orleans, LA, USA, 2016).

